# Glucagon-like Peptide-1 Receptor Agonists and Ocular Disease: Mechanisms, Evidence and Therapeutic Perspectives

**DOI:** 10.3390/ijms27031432

**Published:** 2026-01-31

**Authors:** Xiaoming Gong, Faruk H. Örge

**Affiliations:** 1 Vision Center, Akron Children’s Hospital, Akron, OH 44308, USA; forge@akronchildrens.org; 2 Rebecca D. Considine Research Institute, Akron Children’s Hospital, Akron, OH 44308, USA

**Keywords:** glucagon-like peptide-1 receptor agonists, glaucoma, diabetic retinopathy, age-related macular degeneration, nonarteritic anterior ischemic optic neuropathy

## Abstract

Ocular diseases, including glaucoma, diabetic retinopathy (DR), and age-related macular degeneration (AMD), remain major global causes of irreversible vision loss. Despite advances in clinical management, current therapies insufficiently address the shared metabolic, inflammatory, vascular, and neurodegenerative mechanisms underlying these conditions. Glucagon-like peptide-1 receptor agonists (GLP-1RAs), widely used for type 2 diabetes and obesity, have emerged as multi-target candidates for ocular therapeutics due to their pleiotropic anti-inflammatory, antioxidant, vasculoprotective, and neuroprotective properties. Preclinical studies consistently demonstrate that GLP-1RAs preserve blood–retina barrier integrity, suppress pathological angiogenesis, mitigate oxidative and inflammatory stress, and protect retinal neurons from degeneration. Complementary clinical and real-world evidence shows a robust and reproducible reduction in glaucoma risk among GLP-1RA users across diabetic and non-diabetic populations. By contrast, findings for DR and AMD are more heterogeneous and appear context-dependent, with potential benefits most evident in early or non-exudative disease stages. Emerging safety considerations—including reports of nonarteritic anterior ischemic optic neuropathy and early DR worsening in the setting of rapid glycemic improvement—highlight the need for careful interpretation, individualized risk assessment, and appropriate ophthalmic monitoring. This review synthesizes molecular mechanisms, experimental data, clinical and pharmacoepidemiologic evidence, and safety signals to critically evaluate the therapeutic potential of GLP-1RAs in ocular disease. We also outline key translational challenges, including the need for ocular-targeted delivery strategies, prospective ophthalmology-specific trials, and precision-medicine approaches to determine when and how GLP-1RAs can be safely advanced as disease-modifying treatments in ophthalmology.

## 1. Introduction

Glucagon-like peptide-1 receptor agonists (GLP-1RAs) have rapidly transformed the management of type 2 diabetes mellitus (T2DM) and obesity owing to their robust metabolic, cardiovascular and renal benefits [1]. Beyond their systemic metabolic effects, a growing body of evidence indicates that GLP-1RAs exert potent anti-inflammatory, antioxidant, and neuroprotective actions within ocular tissues, including retinal neurons, Müller glia, retinal pigment epithelium (RPE), and retinal microvascular endothelial cells [2]. These pleiotropic properties, together with the rapidly expanding global use of GLP-1RAs, have generated substantial interest in their potential relevance to ocular diseases such as glaucoma, diabetic retinopathy (DR), and age-related macular degeneration (AMD), in which chronic inflammation, oxidative stress, and neurodegeneration are central drivers of pathology [3].

Clinical observations have increasingly supported this emerging paradigm. Multiple large observational and retrospective cohort studies have reported reduced incidence or slower progression of ocular disease among GLP-1RA users in both diabetic and non-diabetic populations [4,5,6]. Consistent with these findings, the GLP-1 receptor (GLP-1R), a class B1 G protein-coupled receptor, is expressed in multiple human ocular compartments [7,8]. Activation of GLP-1R initiates intracellular signaling cascades, including cAMP/PKA, exchange protein directly activated by cAMP (EPAC-2), ERK1/2, and PI3K/Akt pathways, which collectively attenuate oxidative stress, modulate glial and immune activation, stabilize mitochondrial function, and suppress pro-inflammatory cytokine signaling [9,10]. Additional effects on vascular integrity and extracellular matrix (ECM) remodeling may further contribute to anti-angiogenic and vasculoprotective actions.

Preclinical studies demonstrate that GLP-1RAs promote retinal ganglion cell (RGC) survival, improve mitochondrial homeostasis, and mitigate microglial activation in models of metabolic and neurodegenerative ocular stress [11]. Correspondingly, clinical data suggest reduced risk of glaucoma [12] and potential benefits in DR and AMD [13]. At the same time, emerging concerns regarding adverse ocular events—including reports of nonarteritic anterior ischemic optic neuropathy (NAION) [14,15], early worsening of DR in high-risk individuals undergoing rapid glycemic improvement [16,17], and heterogeneous outcomes in AMD—underscore the need for careful interpretation of the evidence and risk-stratified clinical application.

In this review, we explore the molecular mechanisms through which GLP-1RAs influence ocular pathophysiology, synthesize current preclinical and clinical evidence across major ocular diseases, and integrate mechanistic insights with human data to evaluate the potential of GLP-1RAs as novel disease-modifying therapeutics in ophthalmology. We further address key translational challenges, including drug delivery strategies and safety considerations, that must be resolved before GLP-1RAs can be advanced as multi-targeted ophthalmic therapies.

Although several recent narrative and mini-reviews have examined GLP-1RAs in the context of ocular diseases [18,19,20], much of the existing literature focuses on isolated disease entities or lacks integrated mechanistic-clinical synthesis. To extend prior work, this review provides (i) a more detailed mechanistic framework linking intracellular GLP-1R signaling to disease-specific neurovascular pathology, (ii) an expanded assessment of clinical evidence across a broader spectrum of ocular diseases, including cataract and uveitis, and (iii) a more comprehensive discussion of safety, risk stratification, and translational considerations, including ocular-targeted delivery strategies.

## 2. Search Strategy and Selection Criteria

We conducted a structured literature search of PubMed, Google Scholar, and ClinicalTrials.gov through December 2025 using combinations of the following terms: “GLP-1 receptor agonist,” “ocular disease,” “glaucoma,” “diabetic retinopathy,” “age-related macular degeneration,” “cataract,” “uveitis,” and “optic neuropathy,” along with individual agent names (e.g., semaglutide, liraglutide, exenatide). We included English-language preclinical studies that elucidated relevant molecular and cellular mechanisms, as well as clinical evidence prioritizing randomized controlled trials (RCTs), large observational cohorts, and pharmacoepidemiologic analyses assessing ocular outcomes.

Case reports and smaller series were reviewed selectively when informative for safety signals, particularly rare adverse events such as NAION. This review was not designed as a systematic review; rather, it represents a structured narrative synthesis intended to enhance transparency, contextual interpretation, and balance across mechanistic and clinical evidence.

## 3. Mechanisms of Action of GLP-1RAs in Ocular Tissues

**Figure 1:**
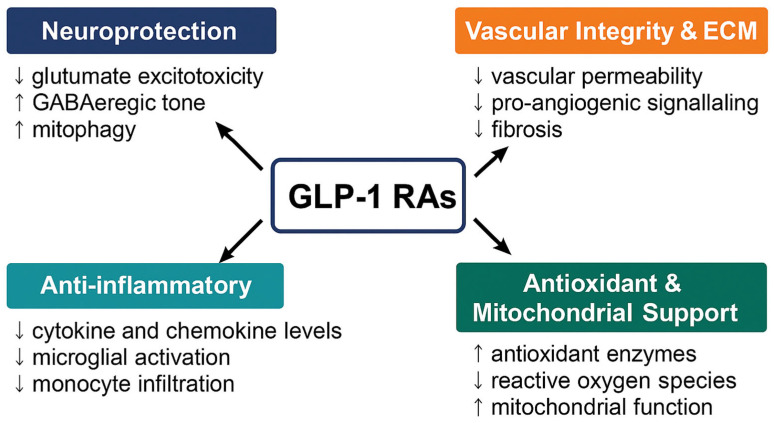
Ocular and systemic mechanisms of GLP-1 receptor agonists (GLP-1RAs). GLP-1RAs exert multifaceted therapeutic effects across four interconnected domains relevant to ocular disease. Neuroprotection (↓ glutamate excitotoxicity, ↑ GABAergic tone, ↑ mitophagy), Anti-inflammatory (↓ TNF-α/IL-1β/IL-6; reduced microglial activation and monocyte infiltration), Antioxidant and Mitochondrial support (↑ SOD/GSH; ↓ ROS; improved mitochondrial integrity), and Vascular integrity and ECM (BRB stabilization; ↓ permeability; restraint of pro-angiogenic signaling; reduced fibrosis). Together, these mechanisms position GLP-1RAs as promising multi-target agents for neurovascular and inflammatory ocular disorders.

GLP-1RAs, including native GLP-1 analogs (albiglutide, beinaglutide, dulaglutide, liraglutide, semaglutide and tirzepatide) and exendin-4-based agents (exenatide and lixisenatide), exert ocular effects primarily through direct activation of GLP-1R expressed on retinal neurons, Müller glia, RPE, vascular endothelial cells, as well as through systemic modulation of inflammation and metabolism [20]. Their detailed molecular mechanisms in ocular tissues are summarized in Table 1, and their actions converge on four interrelated domains relevant to ocular health illustrated in Figure 1.

### 3.1. Neuroprotection

The retina’s exceptional metabolic demand and unique architecture render it highly vulnerable to metabolic (hyperglycemia, obesity), mechanical (elevated IOP), ischemia and age-related stressors. Excitotoxicity, mitochondrial dysfunction, oxidative stress, and glial activation represent shared neurodegenerative pathways across diverse ocular disorders [51]. GLP-1RAs counteract these processes through multiple complementary mechanisms by reducing glutamate-induced excitotoxicity, modulating voltage-gated calcium channel activity, promoting microglia-mediated production of neurotrophic factors and enhancing GABAergic signaling [21]. Exendin-4 increases GABA_A_R-mediated tonic currents in retinal neurons, stabilizing neuronal excitability [22,23], while downstream ERK1/2–HDAC6 signaling supports axonal transport and mitochondrial trafficking, thereby protecting optic nerve and RGC function [24]. Activation of PINK1/Parkin-mediated mitophagy pathway facilitates clearance of damaged mitochondria and supports energy homeostasis [25]. Together, these mechanisms preserve RGC survival, maintain optic nerve integrity and protect visual function in glaucomatous, ischemic and diabetic stress environments.

### 3.2. Vascular Integrity and ECM Homeostasis

GLP-1RAs reinforce blood-retina barrier (BRB) stability and counteract pathological angiogenesis by upregulating tight junction protein expression (occludin, claudin-5) and reducing vascular permeability [26]. These agents indirectly suppress VEGF-driven angiogenesis, thereby limiting neovascularization in proliferative DR and neovascular AMD [27]. Through cAMP/PKA-mediated inhibition of NF-κB signaling, GLP-1RAs downregulate inflammatory and angiogenic gene expression in retinal cells. In Müller glia and RPE, GLP-1R activation reduces reactive gliosis, dampens cytokine release, and restores ECM balance, ultimately mitigating fibrosis [28]. These effects collectively help preserve retinal structure and microvascular homeostasis across DR, ischemic retinopathies and AMD.

### 3.3. Anti-Inflammatory Effects

Chronic activation of microglia and astrocytes drives cytokine/chemokine release, leukostasis and reactive oxygen species (ROS) generation—all major contributors to retinal injury and neurodegeneration [29,39,40,41]. GLP-1RAs exert anti-inflammatory activity through both systemic and local ocular pathways [42]. In individuals with T2DM, GLP-1RA liraglutide treatment reduces circulating inflammatory macrophages and modulates innate immune activity [30]. In the retina, systemic and topical administration of native GLP-1/GLP-1RAs suppresses glial activation in db/db mice [31]. Semaglutide downregulates monocyte chemoattractant protein-1 (MCP-1) and related chemokines, thereby inhibiting monocyte migration and infiltration into ocular tissues [32,33]. GLP-1RAs shift microglia/macrophages from pro-inflammatory (M1)-like toward anti-inflammatory (M2)-like phenotypes, suppressing NF-κB and NLRP3 inflammasome signaling, reducing TNF-α and IL-1β, and enhancing IL-10 production [34,35]. These effects are particularly relevant in glaucoma, where microglial-derived cytokines drive neurotoxic A1 astrocyte formation and BRB disruption [36,37], underscoring the therapeutic relevance of GLP-1RA-mediated immunomodulation. Importantly, many anti-inflammatory actions appear independent of glycemic control and weight loss [38].

### 3.4. Antioxidant and Mitochondrial Support

**Figure 2:**
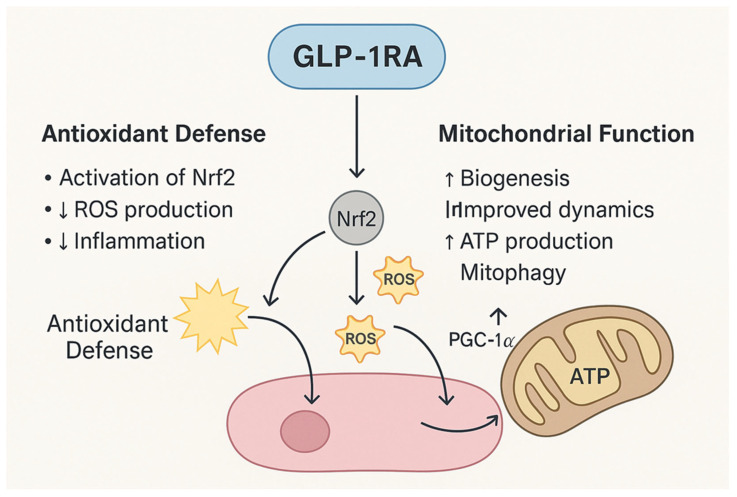
Mechanisms of GLP-1RAs in Antioxidant Defense and Mitochondrial Support. GLP-1RAs exert dual protective effects by reducing oxidative stress and enhancing mitochondrial function. Through activation of Nrf2, GLP-1RAs upregulate antioxidant enzymes, decrease ROS production, and attenuate inflammation. Concurrently, GLP-1RAs stimulate PGC-1α, promoting mitochondrial biogenesis, improving mitochondrial dynamics, enhancing ATP synthesis, and facilitating mitophagy.

Oxidative stress accelerates neuronal apoptosis, vascular dysfunction and RPE damage across DR and AMD. GLP-1RAs provide mitochondrial and antioxidant support through activation of nuclear factor erythroid 2–related factor 2 (Nrf2), a master regulator of antioxidant response, leading to increased expression of antioxidant enzymes such as superoxide dismutase (SOD), catalase, and glutathione peroxidase, while restraining ROS production [43,44]. Concurrently, GLP-1RAs stimulate peroxisome proliferator-activated receptor gamma coactivator 1-alpha (PGC-1α)-driven mitochondrial biogenesis, improve mitochondrial dynamics, and enhance mitophagy [45]. These actions preserve cellular energy homeostasis and reduce oxidative injury, positioning GLP-1RAs as promising candidates for ocular disorders involving mitochondrial dysfunction and mitochondrial compromise (Figure 2).

## 4. Preclinical and Clinical Evidence Across Ocular Diseases

### 4.1. Glaucoma

Glaucoma comprises a heterogeneous group of optic neuropathies characterized by progressive RGC loss, thinning of the retinal nerve fiber layer, and irreversible optic nerve damage. While elevated intraocular pressure (IOP) remains the most important modifiable risk factor, glaucoma pathogenesis is increasingly recognized as a multifactorial process involving neuroinflammation, excitotoxic signaling, mitochondrial dysfunction, vascular dysregulation, and ECM remodeling—pathways that closely overlap with the biological actions of GLP-1RAs.

Extensive preclinical studies support a neuroprotective role for GLP-1RAs in glaucoma. In diabetic and ocular hypertensive models, both systemic and topical administration of agents such as lixisenatide, liraglutide, semaglutide, and the central nervous system–penetrant compound NLY01 have been shown to reduce retinal inflammation, oxidative stress, and glutamate-mediated excitotoxic injury—key contributors to RGC degeneration. Across multiple experimental paradigms, GLP-1RA treatment attenuated microglial and macrophage activation, preserved RGC somata and optic nerve axons, stabilized mitochondrial structure and function, and mitigated pathologic vascular remodeling in diabetic and ocular hypertension models [46,47,48]. These neuroprotective effects are thought to be mediated, at least in part, through cAMP/PKA-dependent suppression of NF-κB signaling, reduced cytokine-driven secondary neurodegeneration, and improved mitochondrial homeostasis. Additionally, activation of nitric oxide-dependent signaling pathways may enhance trabecular meshwork outflow and aqueous humor dynamics, contributing to modest IOP-lowering effects in certain models.

Clinical and real-world evidence increasingly mirrors these preclinical observations. A population-based case–control study involving 1961 individuals demonstrated a significantly lower incidence of glaucoma among GLP-1RA users with T2DM compared with non-users [52]. Multiple retrospective cohort studies have similarly reported reduced rates of open-angle glaucoma in GLP-1RA-treated patients relative to matched control populations [53]. In a large nationwide nested case–control study, GLP-1RA exposure was associated with an approximately 19% reduction in glaucoma onset, with the strongest protective effect observed among individuals receiving therapy for more than three years [54]. Additional real-world datasets have reported lower risks of both glaucoma and ocular hypertension at 1-, 2-, and 3-year follow-up intervals, suggesting that neuroprotective benefits may emerge early and accumulate over time, although heterogeneity in background antidiabetic therapies may contribute to variability across studies [55].

Importantly, evidence of a protective association is not limited to diabetic populations. A large retrospective cohort study of 61,057 overweight or obese nondiabetic individuals demonstrated significantly lower risks of primary open-angle glaucoma (POAG) and ocular hypertension at 3- and 5-year follow-up among GLP-1RA users compared with those prescribed alternative weight-loss medications [56]. A TriNetX-based analysis similarly reported reduced POAG risk among GLP-1RA users compared with individuals treated with metformin, insulin, statins, or aspirin, with a hazard ratio of 0.79 at five years [57]. In that analysis, ocular hypertension incidence was also lower in GLP-1RA users, although absolute IOP reductions were modest and not considered clinically meaningful. Importantly, the protective associations persisted after adjustment for systemic vascular risk factors and concomitant medications, supporting a glaucoma-specific effect beyond general metabolic benefit.

These observations are further supported by a systematic review and meta-analysis of five retrospective studies encompassing more than 156,000 individuals with T2DM, which found a nonsignificant trend toward reduced glaucoma incidence (HR 0.78, 95% CI 0.59–1.04). However, exclusion of a single outlier study yielded a statistically significant reduction (HR 0.71, 95% CI 0.60–0.85) with markedly reduced heterogeneity (I^2^ = 29%) [58].

Although current clinical evidence is largely observational, the consistency of findings across diverse populations and study designs, together with strong mechanistic support from experimental models, reinforces the biological plausibility that GLP-1RAs may act as disease-modifying neuroprotective agents in glaucoma. Future prospective ophthalmology-focused trials incorporating structural and functional endpoints will be essential to determine whether these associations translate into clinically meaningful preservation of vision.

### 4.2. Diabetic Retinopathy (DR)

Diabetic retinopathy arises from chronic hyperglycemia-induced metabolic stress, leading to progressive microvascular degeneration, inflammatory activation, breakdown of the blood-retina-barrier (BRB), neuronal dysfunction, and retinal ischemia. Increasing evidence indicates that retinal neurodegeneration often precedes clinically detectable microvascular lesions, underscoring the need for therapeutic strategies that target early neuronal injury rather than focusing exclusively on late-stage vascular complications. Clinically, DR is broadly classified into an early non-proliferative form (NPDR), characterized by pericyte loss, vascular permeability, capillary nonperfusion, and basement membrane thickening, and an advanced proliferative DR (PDR), defined by pathological neovascularization, vitreous hemorrhage, and tractional retinal detachment. Diabetic macular edema (DME), a vision-threatening complication of both NPDR and PDR, results from BRB disruption and intraretinal fluid accumulation, leading to retinal thickening and visual impairment [59]. By targeting upstream molecular drivers of neurovascular injury and stabilizing BRB integrity, GLP-1RAs have emerged as potential disease-modifying agents in DR beyond their glucose-lowering effects [60].

Preclinical studies across multiple diabetic models have consistently demonstrated that GLP-1RAs exert neuroprotective and vasculoprotective effects in the retina. These agents attenuate retinal neurodegeneration by reducing neuronal apoptosis, enhancing presynaptic γ-aminobutyric acid (GABA) release, and improving synaptic function in RGCs [61]. Exendin-4, for example, has been shown to inhibit voltage-gated calcium channels, thereby promoting RGC survival under diabetic stress conditions [62]. In parallel, GLP-1RA treatment reduces the frequency of pro-inflammatory macrophages and suppresses key chemokines involved in retinal leukostasis, including monocyte chemoattractant protein-1 (MCP-1), CXCL-1/10, and CCL-2. By shifting microglia from a pro-inflammatory M1-like phenotype toward an anti-inflammatory M2-like state through inhibition of NF-κB signaling and NLRP3 inflammasome activation, GLP-1RAs reduce cytokine-mediated injury. Additionally, liraglutide and related agents enhance tight-junction protein expression, reduce vascular permeability, and preserve BRB integrity, thereby limiting edema formation and microvascular damage [63,64]. Together, these findings support the concept that GLP-1RAs act on both neuronal and vascular components of DR and may function as “upstream” therapeutics capable of intervening before irreversible microvascular damage occurs.

Clinical evidence is more heterogeneous, reflecting variability in baseline retinopathy severity, rates of glycemic improvement, treatment duration and patient selection. A meta-analysis of randomized controlled trials (RCTs) reported an increased incidence of early-stage DR events among GLP-1RA-treated patients compared with placebo, yet a reduced risk of progression to advanced DR when compared with insulin therapy, suggesting a context-dependent risk profile [65]. Complementing these findings, Mendelian randomization analyses revealed an inverse association between genetically proxied GLP-1R expression and severe DR, supporting a protective role of GLP-1R signaling in DR pathogenesis [66]. Conversely, a recent retrospective cohort analysis found that concomitant use of GLP-1RAs and insulin was associated with a significantly elevated risk of DR and DME compared with combined SGLT-2 inhibitor and insulin therapy [49]. The SUSTAIN-6 cardiovascular outcomes trial similarly reported a higher incidence of DR-related complications in patients receiving semaglutide compared with placebo (3.0% vs. 1.8%; hazard ratio 1.76), particularly among individuals with pre-existing moderate-to-severe DR and large, rapid reductions in HbA1c. This observation aligns with the well-described phenomenon of “early worsening” of DR that accompanies rapid glycemic improvement with potent glucose-lowering therapies [50,67].

Despite these concerns, multiple large-scale analyses of observational data found no statistically significant association between GLP-1RA use and the exacerbation of DR or the development of sight-threatening retinopathy. Notably, the EXSCEL trial evaluating exenatide reported no significant differences in DR outcomes compared with placebo [68].

Taken together, these findings do not diminish the therapeutic potential of GLP-1RAs in DR but underscore the importance of patient selection, baseline DR stratification, and careful management of glycemic trajectories. Avoiding abrupt glycemic correction and ensuring close retinal monitoring during treatment initiation are particularly important for individuals with established moderate-to-severe DR. With appropriately tailored therapy, longer-term neurovascular benefits may still be realized.

### 4.3. Age-Related Macular Degeneration (AMD)

AMD is a leading cause of irreversible central vision loss in older adults and remains a major unmet clinical need despite the widespread use of anti-VEGF therapies and the recent emergence of complement-based interventions [69,70]. The pathogenesis of AMD is complex and multifactorial, involving chronic inflammation, metabolic dysfunction, mitochondrial impairment, oxidative stress, and ECM remodeling. These interrelated processes contribute to both early non-exudative AMD and progression to advanced forms, including geographic atrophy (GA) and neovascular AMD (nAMD) [71]. Given that GLP-1RAs modulate several of these upstream pathways, interest has grown in their potential role as disease-modifying agents in AMD.

Preclinical evidence, although still limited, supports a biologically plausible role for GLP-1RAs in modulating AMD-relevant mechanisms. A recent study has demonstrated that liraglutide suppresses NLRP3 inflammasome activation and reduces IL-1β release in a laser-induced model of choroidal neovascularization (CNV), resulting in a significant reduction in CNV lesion size [72]. These findings suggest that GLP-1RAs may attenuate innate immune signaling and inflammatory amplification pathways that contribute to angiogenesis, particularly in early or inflammation-driven stages of neovascular disease.

Consistent with these mechanistic insights, several large-scale observational and retrospective cohort studies have reported associations between GLP-1RA use and reduced AMD incidence or slower disease progression. A retrospective cohort study involving nearly 10,000 GLP-1RA users found significantly lower rates of both non-exudative and exudative AMD compared with individuals treated with metformin, insulin, and statins [57]. These findings suggest that the apparent retinal protective effects of GLP-1RAs may extend beyond glycemic control and could reflect direct ocular actions, systemic metabolic benefits, or anti-inflammatory effects. A diabetes-specific cohort study similarly identified significantly reduced AMD onset and progression among GLP-1RA users, supporting the potential disease-modifying effects, particularly in early-stage AMD [73]. In addition, a large TriNetX-based analysis of more than 45,000 non-diabetic overweight or obese individuals found a significantly reduced risk of developing non-exudative AMD over 5- to 10-year follow-up among GLP-1RA users compared with those prescribed other weight-loss drugs; notably, no significant reduction in progression to nAMD was observed [74]. Collectively, these results suggest that GLP-1RAs may be most effective in early or intermediate AMD stages, where inflammatory, metabolic, and mitochondrial dysregulation predominate, but may have more limited impact once angiogenic pathways become dominant.

In contrast, a population-based cohort study using administrative health databases from Ontario, Canada, reported an approximately two-fold higher risk of nAMD among patients with diabetes treated with GLP-1RAs compared with unexposed diabetic individuals [75]. These apparently discordant findings underscore important methodological differences that may account for divergent risk estimates. Although both the TriNetX and Ontario studies applied propensity score matching, critical distinctions remain in comparator selection and outcome definition. In the TriNetX analysis, GLP-1RA users were compared with patients receiving other glucose- and lipid-lowering therapies, whereas the Ontario cohort used untreated diabetic patients as the reference group—raising the possibility of confounding by disease severity and treatment indication. Furthermore, TriNetX captured both non-exudative and exudative AMD across extended follow-up periods, while the Ontario study applied a stringent operational definition of nAMD based on diagnostic coding coupled with initiation of anti-VEGF therapy.

Additional heterogeneity likely arises from differences in baseline glycemic control, AMD stage at treatment initiation, exposure duration, and broader metabolic context. From a mechanistic standpoint, the protective associations observed in TriNetX may reflect improvements in adiposity, lipid metabolism, insulin sensitivity, and chronic inflammation—factors most relevant to early AMD pathogenesis. In contrast, the increased nAMD risk signal observed in the Ontario study could be related to context-dependent pro-angiogenic effects, such as activation of the CXCL12–VEGF signaling axis, or to transient metabolic and hemodynamic stress during rapid glycemic normalization in susceptible individuals. Accordingly, current evidence supports a dual, context-dependent model in which GLP-1RAs may confer protection during early or non-exudative stages of AMD, while potentially facilitating neovascular conversion under specific metabolic or vascular conditions in predisposed patients.

Overall, the clinical landscape for GLP-1RAs in ocular disease remains nuanced and disease-specific. As summarized in Table 2, associations between GLP-1RA exposure and clinical outcomes vary substantially across glaucoma, DR, and AMD, reflecting differences in disease biology, study design, and patient populations. These observations underscore the need for stage-specific mechanistic studies and prospective trials to define precisely where GLP-1RAs may offer therapeutic benefit and where caution is warranted in the management of these ocular diseases.

### 4.4. Cataract

Although cataract formation primarily involves lens-intrinsic protein aggregation and oxidative stress, systemic metabolic and inflammatory states influence risk. GLP-1RAs may reduce cataract development by improving systemic oxidative balance and metabolic stress, reducing chronic inflammation that accelerates lens opacification, and modulating pathways that influence osmotic and glycation-related lens damage. A recent large retrospective cohort study leveraging the TriNetX Global Collaborative Network reported significantly reduced risk of age-related cataract among nondiabetic overweight/obese individuals treated with GLP-1RAs compared with other weight-loss medications or no pharmacotherapy [76]. Similar protective trends appear in T2DM populations, although prospective validation is needed. Given the long-time course of cataract development and the favorable safety profile of GLP-1RAs, prevention studies may be feasible.

### 4.5. Uveitis

Uveitis encompasses a diverse group of intraocular inflammatory conditions that can cause irreversible vision loss through persistent cytokine-mediated tissue injury, macular edema, and secondary glaucoma [77]. Non-infectious uveitis is strongly associated with dysregulated T-cell responses and aberrant innate immune activation. Emerging real-world evidence suggests that GLP-1RA use can reduce the risk of developing non-infectious uveitis. A large TriNetX-based cohort study reported a 51.7% relative reduction in risk of developing a new diagnosis of uveitis among GLP-1RA users compared with matched non-users in both diabetic and non-diabetic populations [78]. Compared with other anti-diabetic agents, GLP-1RAs conferred greater protection than metformin and insulin, though slightly less than SGLT2 inhibitors. These data support further exploration of GLP-1RAs as systemic adjuncts in inflammatory ophthalmic diseases.

## 5. Safety Considerations

As the use of GLP-1RAs continues to expand for diabetes, obesity, and cardiometabolic indications, several ophthalmic safety signals have emerged from observational studies and pharmacovigilance datasets. Although such adverse events (AEs) remain rare, patterns across independent data sources warrant careful clinical attention. Importantly, the overall benefits of GLP-1RAs must be weighed against these potential risks, particularly in patients with pre-existing ocular vulnerability [79,80,81].

### 5.1. Nonarteritic Anterior Ischemic Optic Neuropathy (NAION)

Among reported ocular AEs, NAION has received the greatest scrutiny, particularly in relation to semaglutide exposure. Several real-world datasets have suggested an association between semaglutide use and increased NAION incidence; however, it is critical to interpret these findings within the context of study design limitations and absolute risk, as NAION remains a rare clinical outcome.

A neuro-ophthalmology referral-registry cohort reported significantly higher relative risk of NAION among semaglutide users compared with matched non-GLP-1RA controls, with hazard ratios (HRs) of 4.28 (95% CI 1.62–11.29) in individuals with T2DM and 7.64 (95% CI 2.21–26.36) in patients with obesity or overweight [82]. Despite these elevated relative hazards, the absolute number of events was small, reflecting the low baseline incidence of NAION in the general population. In a large retrospective analysis spanning 14 databases (six administrative claims datasets and eight electronic health record systems), semaglutide use among individuals with T2DM was associated with an incidence rate of approximately 14.5 NAION events per 100,000 person-years, compared with substantially lower background rates in matched comparator groups [83]. Similarly, Danish and Norwegian register-based studies reported an approximately two-fold increase in relative NAION risk among semaglutide users; however, absolute incidence remained on the order of a few additional cases per 100,000 treated individuals per year, prompting regulatory review by the European Medicines Agency (EMA) and the World Health Organization (WHO) [84,85].

Consistent with these observations, Pharmacovigilance analyses from the FDA Adverse Event Reporting System (FAERS) and VigiBase identified disproportionate reporting of NAION with semaglutide compared with other glucose-lowering medications [86,87,88], but these spontaneous reporting systems cannot provide valid incidence estimates and are inherently vulnerable to reporting bias, stimulated reporting, incomplete clinical data, and confounding by indication.

Conversely, a large multinational population-based study using the TriNetX global health research network, which included individuals with T2DM, obesity or both, found no significant increase in the risk of NAION among semaglutide users compared with those receiving other glucose-lowering or weight-loss medications across multiple subgroups and at 1-, 2-, and 3-year follow-up intervals [89]. In this analysis, absolute NAION incidence remained low across all treatment cohorts, reinforcing the overall rarity of the event at the population level. These discrepant findings underscore critical methodological differences across studies, including comparator selection (active vs. untreated controls), outcome definitions, duration of follow-up, and the degree of adjustment for key vascular and ophthalmic risk factors.

From a mechanistic perspective, NAION is a multifactorial condition influenced by vascular risk factors, optic disk anatomy (so-called “disk-at-risk”), obstructive sleep apnea, nocturnal hypotension and systemic hemodynamic changes. The potential contribution of rapid metabolic shifts—such as abrupt reductions in blood pressure, glycemia or intravascular volume—remains an area of active investigation and may partially explain context-dependent risk signals observed in some cohorts. Importantly, all currently available evidence derives from observational studies and pharmacovigilance databases, precluding causal inference.

Taken together, existing data suggest that even if an association between semaglutide and NAION exists, the absolute risk to individual patients is very low. Nevertheless, given the severity of vision loss associated with NAION, these findings support the need for heightened ophthalmic vigilance, risk-informed monitoring in susceptible individuals (e.g., those with known optic nerve vulnerability or multiple vascular risk factors), and cautious interpretation as GLP-1RA use continues to expand across broader and healthier populations.

### 5.2. Other Retinal and Optic Nerve Events

Beyond NAION, pharmacovigilance and real-world data have identified signal enrichment for other rare ocular AEs associated with GLP-1RA exposure, including retinopathy exacerbation, retinal detachment, vitreous hemorrhage and optic nerve disorders [90]. As with NAION, these signals are characterized by very low absolute event rates, typically corresponding to only a few cases per 10,000–100,000 person-years, depending on the outcome and population studied. Although these events remain uncommon, their appearance across multiple independent datasets warrants continued post-marketing surveillance. Ongoing monitoring is particularly important as GLP-1RAs are increasingly prescribed to larger and metabolically healthier populations, in whom baseline ocular risk may be lower but cumulative exposure durations may be longer. Framing these safety signals in absolute terms is essential to avoid overestimation of clinical risk while ensuring appropriate vigilance in susceptible patients.

### 5.3. Conflicting Evidence and Null Findings

Despite these concerning signals, several large and well-designed epidemiologic studies have not consistently replicated the elevated NAION risk. A large multinational population-based study found no significant increase in the risk of NAION among semaglutide users compared to those receiving other glucose-lowering or weight-loss medications [89]. A comprehensive systematic review and meta-analysis similarly concluded that the overall incidence of ocular AEs with semaglutide remains low, and that current evidence is insufficient to confirm a direct causal relationship [91]. These discrepancies highlight the limitations of current data, including heterogeneity in baseline vascular risk, DR severity, follow-up duration, and confounding by indication. Overall, current evidence supports a cautious but not prohibitive approach to GLP-1RA use from an ophthalmic safety perspective.

### 5.4. Clinical Implications

Given emerging but inconclusive data, clinicians should adopt a risk-informed monitoring strategy when prescribing GLP-1RAs, especially semaglutide. Patients, particularly those with risk factors such as pre-existing optic nerve vulnerability, severe sleep apnea, or vascular risk factors, may benefit from baseline ophthalmic evaluation prior to initiation of semaglutide or similar agents. Symptoms such as sudden visual field loss, dyschromatopsia, or decreased visual acuity should prompt urgent ophthalmic referral. Close retinal monitoring should be performed during periods of rapid metabolic improvement in patients with moderate to severe diabetic retinopathy, as accelerated glycemic correction is associated with the well-characterized phenomenon of early DR worsening. As global use of GLP-1RAs continues to grow and expand to younger, healthier populations, prospective ophthalmology-specific studies and robust post-marketing surveillance will be essential for identifying true risk, clarifying mechanisms, and guiding evidence-based safety recommendations.

## 6. Challenges and Perspectives

Although GLP-1RAs show substantial promise as multi-target therapeutics for ocular disorders, several scientific, clinical, and translational challenges must be addressed before they can be reliably incorporated into routine ophthalmic practice. The following subsections outline key knowledge gaps and future directions necessary to fully realize the therapeutic potential of GLP-1RAs in vision science.

### 6.1. Need for Targeted Ocular Delivery Strategies

To date, the majority of preclinical and clinical studies investigating the ocular effects of GLP-1RAs have relied on systemic administration for cardiometabolic indications. While this approach has provided important real-world insights, it complicates interpretation by making it difficult to disentangle direct ocular actions from indirect benefits mediated by improvements in glycemic control, systemic inflammation, body weight, or cardiovascular function. Moreover, systemic delivery limits drug bioavailability within ocular tissues and exposes patients to off-target effects—most notably gastrointestinal intolerance—thereby constraining dose optimization for eye-specific indications.

From a pharmacologic perspective, GLP-1RAs are relatively large peptide molecules with limited penetration across the blood–retina barrier, further restricting tissue-specific exposure following systemic dosing. These properties underscore a critical translational challenge: without ocular-targeted delivery, it remains difficult to define the true retinal therapeutic index, dose–response relationship, and mechanism of action of GLP-1RAs within the eye. Consequently, the development of ocular-targeted delivery strategies represents a key inflection point for advancing GLP-1RAs from systemically administered agents with secondary ocular benefits to bona fide ophthalmic therapeutics.

Future research should therefore prioritize localized and sustained intraocular delivery approaches designed to achieve therapeutically relevant ocular concentrations while minimizing systemic exposure. Potential strategies include topical formulations designed for enhanced corneal or conjunctival penetration; intravitreal administration to directly target retinal neurons, glia, and vasculature; sustained-release systems such as injectable depots, microspheres, or biodegradable implants; and viral vector–based gene therapies capable of providing prolonged, tissue-specific GLP-1R activation. Collectively, these approaches could substantially reduce systemic confounding, limit off-target adverse effects, and enable more rigorous evaluation of GLP-1RA-mediated neuroprotective, anti-inflammatory, and vasculoprotective effects within ocular tissues.

### 6.2. Need for Rigorous Prospective Clinical Trials

Current evidence supporting the potential ocular benefits of GLP-1RAs is derived predominantly from retrospective cohort studies, real-world databases, or post hoc analyses of large systemic trials that were not originally designed with ophthalmic endpoints in mind. Although these studies provide important hypothesis-generating insights, they are inherently limited by residual confounding, heterogeneous outcome definitions, and lack of standardized ocular assessments. To establish GLP-1RAs as bona fide disease-modifying agents in ophthalmology, carefully designed prospective clinical trials with ophthalmology-specific objectives and endpoints are urgently needed.

Such trials should prioritize structural endpoints that are sensitive to early disease modification, including retinal nerve fiber layer (RNFL) and ganglion cell–inner plexiform layer thickness on optical coherence tomography (OCT) for glaucoma, and retinal thickness, neurodegenerative markers, and microvascular metrics for DR and AMD. Structural endpoints offer higher reproducibility and statistical power than traditional visual acuity measures and should serve as primary endpoints, particularly in early-stage disease. Functional outcomes—such as visual field testing, electroretinography (ERG), and contrast sensitivity—should be incorporated as secondary or exploratory endpoints to assess clinical relevance over longer follow-up periods.

Assessment of vascular integrity and inflammatory activity should be systematically integrated using OCT angiography (OCT-A) and fluorescein angiography to quantify capillary nonperfusion, vascular leakage, and BRB disruption. Parallel measurement of circulating and intraocular biomarkers of neurodegeneration, oxidative stress, and inflammation may further strengthen mechanistic interpretation and aid patient stratification. Sample size estimation should be driven by expected rates of structural change rather than incident disease endpoints, enabling feasible enrollment and shorter study duration. Follow-up periods should be sufficient to capture both early neuroprotective signals (e.g., 12–24 months for structural measures) and longer-term disease progression or stabilization.

Importantly, prospective trials should include dose–response and exposure-duration analyses, particularly given the context-dependent effects observed in existing studies. Comparative evaluation of different GLP-1RA subclasses, dosing intensities, and delivery strategies will be necessary to determine whether ocular effects represent a class-wide phenomenon or are agent-specific. Such trials will be essential to move the field beyond associative signals toward causal, mechanism-driven evidence.

### 6.3. Safety Evaluation and Risk Stratification

As GLP-1RA use expands across metabolic, cardiovascular, and weight-management indications, a more refined approach to ocular safety evaluation and patient-level risk stratification is essential. Emerging safety considerations—including “early worsening” of DR during rapid glycemic improvement and observational signals linking semaglutide use with NAION—highlight the importance of contextual, risk-informed clinical decision-making rather than a one-size-fits-all approach.

Future studies should aim to identify and validate specific risk domains that contribute to adverse ocular outcomes. These include (i) baseline ocular disease severity (e.g., moderate-to-severe DR, crowded optic disks), (ii) glycemic trajectories, particularly the rate and magnitude of HbA1c decline, (iii) systemic vascular risk factors such as hypertension, dyslipidemia, and atherosclerotic disease, and (iv) non-metabolic contributors including severe obstructive sleep apnea, nocturnal hypotension, and autonomic dysfunction. Incorporating these variables into multivariable risk models would enable more individualized risk prediction.

Equally important is the development of standardized ophthalmic monitoring protocols tailored to baseline risk. High-risk individuals—such as patients with advanced DR or known optic nerve vulnerability—may benefit from baseline ophthalmic evaluation prior to GLP-1RA initiation and closer follow-up during periods of rapid metabolic change. Lower-risk individuals may require less intensive surveillance, thereby preserving clinical resources while maintaining patient safety. Clear guidance linking risk strata to monitoring frequency and management strategies will be critical for real-world implementation.

Comparative safety assessments across individual GLP-1RAs, dual incretin agonists (e.g., tirzepatide), and emerging multi-agonist therapies should be prioritized, as differences in potency, pharmacokinetics, and metabolic effects may translate into distinct ocular risk profiles. Ultimately, adoption of a precision-medicine framework—integrating systemic metabolic variables with ocular structural and functional characteristics—will be necessary to maximize the therapeutic potential of GLP-1RAs while minimizing the likelihood of vision-threatening adverse events.

### 6.4. Implications for the Ophthalmic Community

As GLP-1RA use continues to expand globally for metabolic, cardiovascular, and weight-management indications, ophthalmologists are uniquely positioned to define their role as potential disease-modifying agents in eye care. Progress in this area will require integration of molecular insights from retinal biology and neuroinflammation with advanced ocular imaging, functional testing, and real-world evidence derived from large health-system datasets.

By combining mechanistic understanding with longitudinal clinical data and patient-specific risk stratification based on both systemic and ocular characteristics, the ophthalmic community can help identify which patient populations and disease stages are most likely to benefit from GLP-1RA therapy. If these translational challenges are effectively addressed, GLP-1RAs may ultimately emerge as a new class of multimodal therapeutics targeting shared neurovascular and inflammatory pathways that underlie many of the leading causes of irreversible vision loss.

## 7. Conclusions

GLP-1RAs are emerging as compelling multi-target therapeutic candidates for ocular diseases, owing to their convergent actions on neuroinflammation, mitochondrial dysfunction, oxidative stress, and vascular instability. Across glaucoma, DR, AMD, and other retinal and optic nerve disorders, both preclinical and clinical evidence consistently points to protective effects on neuronal survival, vascular integrity, and inflammatory responses. These benefits appear to extend beyond glycemic control, suggesting a broader potential for GLP-1RAs as disease-modifying agents in ophthalmology.

Despite this encouraging trajectory, important challenges remain. Current clinical data are derived largely from retrospective analyses or systemic trials not designed with ophthalmic endpoints in mind, making it difficult to disentangle direct ocular effects from systemic metabolic improvements. Additionally, emerging safety considerations—such as early DR worsening in patients with rapid glycemic correction and rare optic nerve events including NAION—underscore the need for careful patient selection, structured monitoring, and mechanistically informed risk stratification.

Looking ahead, the development of ocular-targeted delivery methods, combined with rigorously designed prospective clinical trials incorporating structural, vascular, inflammatory, and functional endpoints, will be essential for determining the true therapeutic potential of GLP-1RAs in ocular disease.

With continued multidisciplinary investigation and thoughtful clinical integration, GLP-1RAs have the potential to inaugurate a new class of therapeutics aimed at the shared neurovascular and inflammatory mechanisms that contribute to the most common causes of irreversible vision loss worldwide.

## Figures and Tables

**Table 1:** Summary of Molecular Mechanisms of GLP-1RAs in Ocular Tissues.

Mechanisms of Actions	Ocular Targets	Molecular Pathways	Molecular Effectors	Functional Outcomes	Ocular Disease	GLP-1 RAs	Ref.
Neuroprotection (RGC survival)	RGCs, Amacrine cells	GLP-1R→cAMP/ PKA, PI3K/Akt, ERK1/2; modulation of Ca^2+^ channels; GABAergic tone	↑ BDNF signaling; ERK1/2–HDAC6 axis (axonal transport); PINK1/Parkin mitophagy;	Resilience to excitotoxic/ ischemic stress; preserved axoplasmic flow	Glaucoma (RGC loss), optic neuropathy, DR	Exenatide, liraglutide, semaglutide	[21,22,23]
Mitochondrial quality control	RGCs, photo- receptors	AMPK→PINK1/ Parkin; Mitophagy; ERK1/2–HDAC6	↑ Mitophagy; ↓ damaged mitochondria; stabilized mitochondrial trafficking;	Sustained ATP; ↓ ROS-induced apoptosis	Glaucoma, DR, AMD	Exenatide, liraglutide, semaglutide	[24,25]
Endothelial barrier stabilization	Retinal vascular endothelium; pericytes	PI3K/Akt; Rac1/cytoskeletal junctions	↑ Tight-junction proteins (occludin/ claudin-5); ↓ leukostasis	Preserved BRB; ↓ vascular leakage and edema	DME; ischemic retinopathies	Liraglutide, dulaglutide	[26,27]
Anti-angiogenic responses	Endothelium; RPE	Indirect VEGF modulation; HIF-1α restraint	↓ VEGF/VEGFR signaling; ↓ endothelial proliferation	↓ Pathologic neovascularization	PDR, nAMD	Class	[28,29]
Microglial modulation	Microglia, Müller glia	cAMP/PKA; NF-κB and NLRP3 inhibition	↓ TNF-α, IL-1β, IL-6; microglial deactivation	↓ Neuro- inflammation; ↓ secondary neuronal damage	DR, AMD, glaucoma	Class	[30,31,32,33,34,35,36,37,38]
Immune modulation	Uveal tract, choroid	Systemic + local antiinflammation	↓ Leukocyte recruitment	↓ Risk of ocular inflammation	Uveitis (non- infectious)	Class	[39,40,41,42]
Antioxidant defense	Neurons; Endothelium; RPE	AMPK/Nrf2 activation	↑ SOD, ↑ GSH; ↓ NADPH oxidase activity	↓ ROS load; Protection from hyperglycemia- induced oxidative stress	DR, AMD	Class	[43,44,45]
Neurovascular coupling and IOP control	TM/uveoscleral outflow; optic nerve head	cAMP signaling; nitric-oxide (NO) pathways	Potential outflow enhancement; vascular autoregulation	↓ IOP (in some reports); optic nerve perfusion support	Glaucoma	Class	[46,47,48]
Systemic metabolic context	Retina, Choroid	Rapid HbA1c reduction; BP/volume shifts	Transient perfusion stress	Early worsening of DR in vulnerable eyes	DR safety concerns	Class	[49,50]

Abbreviations: AMPK, AMP-activated protein kinase; BDNF, brain-derived neurotrophic factor; BP, blood pressure; BRB, blood retina barrier; DME, diabetic macular edema; DR, diabetic retinopathy; GSH, glutathione; IOP, intraocular pressure; nAMD, neovascular AMD; NF-κB, nuclear factor kappa-light-chain-enhancer of activated B cells; NLRP3, NOD-, LRR- and pyrin domain-containing protein 3; PDR, proliferative DR; RGC, retinal ganglion cell; SOD, superoxide dismutase; TM, trabecular meshwork; VEGF, vascular endothelial growth factor; ↑ upregulation; ↓ downregulation.

**Table 2:** Current Clinical Studies and Trials of GLP-1RAs’ Effects on Glaucoma, Diabetic retinopathy, and AMD.

Key Studies	Population	Study Design	GLP-1RA(s)	Exposure Duration	Primary Ocular Outcome(s)	Main Findings	Limitations
Sterling et al., 2023 [52]	T2DM	Population Case–control	Class	Not reported	Incident glaucoma	Reduced risk	Residual confounding
Chuang et al., 2024 [53]	T2DM	Retrospective cohort	Class	Not reported	OAG incidence	Reduced risk vs. non-users	Medication switching;
Niazi et al., 2024 [54]	T2DM	Nested Case–control	Class	>3 yrs	Glaucoma onset	~19% relative risk reduction	Indication bias; registry limits
Muayad et al., 2025 [55]	T2DM	Comparative cohort	Class vs. metformin	1–3 yrs	Glaucoma risk	Reduced risk at 1, 2, 3 years	Metabolic differences
Vasu et al., 2025 [56]	Non-diabetic obese	Retrospective cohort	Class	3–5 yrs	POAG/OHT incidence	Significant reduction	Lifestyle confounders
Kapoor et al., 2023 [65]	T2DM	Meta-analysis of RCTs	Class	Variable	DR progression	↑ early DR vs. placebo; ↓ vs. insulin	Heterogeneity in DR severity
SUSTAIN-6 (Marso et al., 2016) [16]	T2DM	RCT	Semaglutide	2 yrs	DR complications	HR 1.76 (higher risk with rapid HbA1c decline)	Rapid glycemic reduction; high-risk DR
EXSCEL (Bethel et al., 2020) [68]	T2DM	RCT	Exenatide	3.2 yrs	DR progression	No difference vs. placebo	Not designed for retinal endpoints
Talebi et al., 2025 [4]	T2DM	Real world cohort	Class	Multi-year	DR worsening	↓ progression after adjusting for HbA1c	Glycemic improvement bias
Tauqeer et al., 2025 [6]	T2DM	Observational cohort	Class	Not reported	Progression to VTDR	No increased risk	Limited imaging verification
Zheng et al., 2023 [66]	T2DM	Mendelian randomization	GLP-1R expression	—	Severe DR	Inverse association	Proxy limits
Allan et al., 2025 [57]	Mixed diabetic and non-diabetic	Observational cohort	Class	Multi-year	Non-exudative and exudative AMD	Reduced onset across AMD	Confounding by comorbidity
McLaughlin et al., 2025 [73]	T2DM	Registry cohort	Class	Multi-year	AMD onset and progression	↓ onset; ↓ progression	Diabetes restricted cohort
Ahuja et al., 2025 [74]	Non-diabetic overweight or obese	TriNetX cohort	Class	5–10 yrs	Non-exudative AMD	Long-term reduction	No effect on conversion to nAMD
Shor et al., 2025 [75]	Mixed populations	Registry analysis	Class	Not reported	Neovascular AMD (nAMD)	Mixed findings; some null	Baseline AMD heterogeneity

Abbreviations: AMD, age-related macular degeneration; DR, diabetic retinopathy; nAMD, neovascular AMD; OAG, open angle glaucoma, OHT, ocular hypertension; POAG, primary open-angle glaucoma; VTDR, vision-threatening diabetic retinopathy. ↑ increased; ↓ reduced.

## Data Availability

No new data were created or analyzed in this study. Data sharing is not applicable to this article.
